# Role of taurine on acid secretion in the rat stomach

**DOI:** 10.1186/1423-0127-18-11

**Published:** 2011-02-05

**Authors:** Kai-Han Huang, Chia-Chieh Chang, Jau-Der Ho, Ruey-Hwa Lu, Li Hsueh Tsai

**Affiliations:** 1 Department of Ophthalmology, Taipei Medical University Hospital, Taipei 11031, Taiwan; 2 Graduate Institute of Clinical Medicine, College of Medicine, Taipei Medical University, Taipei 11031, Taiwan; 3 Graduate Institute of Medical Sciences, College of Medicine, Taipei Medical University, Taipei 11031, Taiwan; 4 Department of General Surgery, Taipei City Hospital, Taipei 10341, Taiwan; 5 Department of Physiology, School of Medicine, College of Medicine, Taipei Medical University, Taipei 11031, Taiwan

## Abstract

**Background:**

Taurine has chemical structure similar to an inhibitory neurotransmitter, γ-aminobutyric acid (GABA). Previous studies on GABA in the stomach suggest GABAergic neuron is involved in acid secretion, but the effects of taurine are poor understood.

**Methods:**

The effects of taurine on acid secretion, signal transduction, and localization of taurinergic neurons were determined in the rat stomach using everted whole stomach, RIA kit and immunohistochemical methods.

**Results:**

We used antibodies against taurine-synthesizing enzyme, cysteine sulfuric acid decarboxylase (CSAD), and taurine. CSAD- and taurine-positive cells were found in the muscle and mucosal layers. Distributions of CSAD- and taurine-positive cells in both mucosal and muscle layers were heterogeneous in the stomach. Taurine at 10^-9^~10^-4 ^M induced acid secretion, and the maximum secretion was at 10^-5 ^M, 1.6-fold higher than the spontaneous secretion. Taurine-induced acid secretion was completely inhibited by bicuculline and atropine but not by cimetidine, proglumide, or strychnine. Atropine and tetrodotoxin (TTX) completely inhibited the acid secretion induced by low concentrations of taurine and partially inhibited induced by high concentrations. Verapamil, a calcium blocker agent, inhibited acid output elicited by taurine. We assumed all Ca^2+ ^channels involved in the response to these secretagogues were equally affected by verapamil. Intracellular cAMP (adenosine 3', 5'-monophosphat) in the stomach significantly increased with taurine treatment in a dose-dependent manner. High correlation (*r*=0.859, *p *< 0.001) of taurine concentrations with cAMP was observed.

**Conclusions:**

Our results demonstrated for the first time in taurine-induced acid secretion due to increase intracellular calcium may act through the A type of GABA receptors, which are mainly located on cholinergic neurons though cAMP pathway and partially on nonneuronal cells in the rat stomach.

## Background

Inhibitory amino acids (IAAs), e.g., taurine and γ-aminobutyric acid (GABA), are present in various parts of the vertebrate central nervous system (CNS) and serve as major inhibitory neurotransmitters [1]. Taurine is the most abundant free amino acid in the body and is present at high concentrations during development. It is synthesized from cysteine via oxidation of cysteine to cysteinesulfinate by the enzyme cysteine dioxygenase (CDO), followed by the decarboxylation of cysteinesulfinate to hypotaurine, catalyzed by cysteine sulfuric acid decarboxylase (CSAD) [2,3].

Taurine has many physiological properties, including membrane stabilization, osmoregulation, neuromodulation, regulation of calcium homeostasis, antioxidation, modulation of ion flux, and serving as a neurotransmitter or neuromodulator [4-8].

Taurine has chemical structure similar to an inhibitory neurotransmitter GABA which binds to GABA_A_, GABA_B_, and the glycine receptor [9-12]. It protected the gastric mucosa against certain lesions [13-16]. Taurine is stored in parietal cells [17] and smooth muscle [18]. It plays an import role in stabilizing membranes [5], and modulating acid secretion and gastric motility.

Studies on GABA in the enteric nervous system suggested that GABAergic neurons are not confined to the CNS, but rather these neurons also exist in the peripheral autonomic nervous system [19-21] and are involved in acid secretion [22] and motility [23]. However, the functions of taurine in gastric secretion are largely unknown. Recently, pharmacological studies have found that taurine binds to GABA receptors [24-26]. The purpose of the study was to determine if taurine also regulates gastric acid secretion via GABA receptors in the stomach.

Localization of taurine in the CNS used enzymatic synthesis of CSAD enzymes [10,11]. CSAD forms antibodies in the hippocampus, cerebellum, and retina [27-29]. However, no detailed information is available for the stomach.

In this communication, we demonstrated that taurine might regulate acid secretion through A- type GABA receptors and elevation of cAMP in the stomach. The distribution of taurine-containing cells in the rat stomach was localized immunohistochemically using specific antibodies against taurine and CSAD.

## Methods

### Chemical and antibodies

Taurine, bicuculline, cimetidine, proglumide, atropine, strychnine, tetrodotoxin (TTX), verapamil, and 3-isobutyl-1-methylxanthane (IBMX) were purchased from Sigma Chemical (St. Louis, MO, USA). The [^3^H] cAMP (adenosine 3', 5'-monophosphat) assay system was obtained from Amersham (Buckinghamshire, UK). Anti-taurine was purchased from Abcam (Cambridge, UK). Anti-CSAD was a gift from Dr. Wu, J-Y (Department of Biomedical Science, Florida Atlantic University, Boca Raton, Florida 33431, USA). Other chemicals used were of reagent grade and were obtained from various commercial sources.

### Animals

Male Sprague-Dawley rats (National Laboratory Animal Center, Taipei, Taiwan) weighing 180~250 g were used. They were housed in group cages under controlled illumination (light cycle, 08:00~20:00), relative humidity of 30%~70%, and temperature (23 ± 1°C) with free access to a laboratory diet (LabDiet, Brentwood, MO, USA) and tap water. Approval for the study was obtained from the Animal Care and Use Committee of Taipei Medical University.

### Immunohistochemical Procedures

The immunohistochemical procedures were described in detail elsewhere [30]. Briefly, male Sprague-Dawley rats were initially anesthetized with an intraperitoneal injection of sodium pentobarbital (50 mg/kg), followed by perfusion with 1 L saline at 37°C, and subsequent fixation with 4% paraformaldehyde in phosphate-buffered saline (PBS: 50 mM potassium phosphate buffer (pH 7.4) containing 0.9% NaCl) at 4°C. After fixation, the tissue was frozen, embedded in OTC compound, mounted on a gelatinized slide, and sectioned at 20~30 μm. The body and antrum of the stomach were used for immunohistochemical studies by the peroxidase-antiperoxidase (PAP) technique [31]. Tissue sections were treated in the following manner: (i) incubated with anti-CSAD (1:300) or anti-taurine (1:1000; Abcam) (diluted in 0.1 M PBS containing 0.1% Triton X-100) for 16 h at 4°C; (ii) rinsed twice with 0.1 M PBS; (iii) incubated in PAP solution (at a 1:50 dilution) in 50 mM Tris-HCl (pH 7.6) for 2 h at room temperature; (iv) rinsed with 50 mM Tris-HCl (pH 7.6) twice; (v) incubated in a solution containing 0.05% diaminobenzidine and 0.01% H_2_O_2 _in 50 mM Tris-Cl (pH 7.6), for 8~10 min at room temperature; and (vi) the sections were dehydrated, mounted on slides with Permount (Fisher), and covered with cover slips for light-microscopic examination. For control experiments, sections were treated exactly as those described above for the experimental group except that antibodies had been preabsorbed with an excess of respective antigens and then were used to replace the anti-taurine or anti-CSAD. Anti-CSAD as described elsewhere [27]. Taurine-containing cells were determined by using specific antibodies from Abcam. For the control experiments anti-taurine and anti-CSAD sera were replaced with preimmune rabbit serum at the same dilution.

### Experiments on Everted Whole Stomachs

Experiments on everted whole stomachs were performed as described elsewhere [30], with slight modifications. Briefly, male Sprague-Dawley rats (weighing 180~250 g) were deprived of food overnight, and allowed free access to water to ensure that the stomach was free of solid contents. A rat was decapitated, and its stomach was immediately removed. The entire everted organ was then placed in a 20-ml organ bath containing a mucosal saline solution (in mM: NaCl, 119; KCl, 4.7; CaCl_2_, 2.5; and glucose, 5.6; pH 5.2) at 30 ± 1°C and continuously bubbled with 100% O_2_. The serosal side was perfused with a serosal saline solution (in mM: NaCl, 119; KCl, 4.7; CaCl_2_, 2.5; NaHCO_3_, 25; KH_2_PO_4_, 1.03; and glucose, 5.6; pH 7.4) at a rate of 1 ml/min under the same conditions as described above except that 100% O_2_ was replaced by a mixture of 95% O_2_ and 5% CO_2_. One hour after equilibration of the organ, the mucosal saline solution was replaced every 15 min during the experiment. Only the serosal side of the preparation was exposed to the test drugs.

Spontaneous acid secretion was determined for 60 min before adding the test drugs. Acid secretion was allowed to last for an additional hour. The acid accumulated on the mucosal side was initially titrated to pH 5.2 and pH 7.0 with 0.1 mM NaOH. Responses of the stomach to drug treatments were expressed as the secretory ratio (R), which was defined as:

R = (secretion evoked by the drug)/(average spontaneous secretion).

The average spontaneous secretion was calculated using acid from the four periods immediately before exposure to the test drugs. Finally, the secretory ratio at the peak response was measured to assess the concentration-response curves.

### Measurement of the cAMP Concentration

Stomachs were cut into 0.4 × 0.4-mm cubes with a Mellwain tissue chopper. After preincubation in a serosal saline solution containing 0.5 mM IBMX maintained at 37°C and continuously bubbled with 95% O_2_ and 5% CO_2_. They were incubated in medium containing 0.5 mM IBMX for 30 min. The mixture was incubated 2 min in the presence or absence of different doses of taurine (10^-9^~10^-4 ^M) according to protocols provided by the supplier (RPA 538; Amersham Biosciences).

After incubation, tissues were homogenized in 6% trichloroacetic acid, followed by centrifugation at 3,000 *g *and 4°C for 15 min. The supernatant was neutralized to pH 7.4 with 1 M Tris, followed by extraction with ether four times. The ether extracts were combined and dried. The cAMP concentration was determined using a commercial RIA kit. The homogenized solution was solubilized in 3 N NaOH and used for protein determination as previously described [32].

### Statistical Analysis

Results are expressed as the mean ± SEM (*n *= sample number). Data were analyzed by Dunnett's test or Student's *t*-test; a *p *value of ≤ 0.05 was considered statistically significant.

## Results

### Immunohistochemical Studies

**Figure 1:**
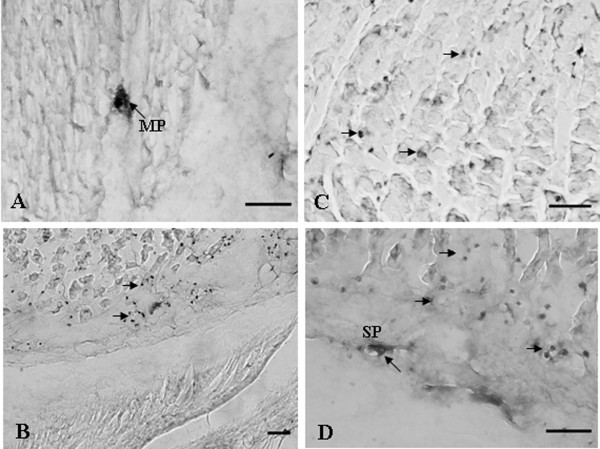
**Immunohistochemical localization of cysteine sulfuric acid decarboxylase (CSAD) in the rat stomach**. (A) Light micrography of a transverse section of the muscle layer showing CSAD-immunoreactive processes in the Body. (B) Light micrograph of cross section showing CSAD-positive processes in the antrum. (C) CSAD-immunoreactive cells occurred mostly in glands of the gastric mucosa. (D) CSAD-positive cell processes in the deep of mucosal layers. MP, myenteric plexus; SP, submucosa plexus. Arrowheads indicate CSAD-positive processes. Bar = 50 μm.

**Figure 2:**
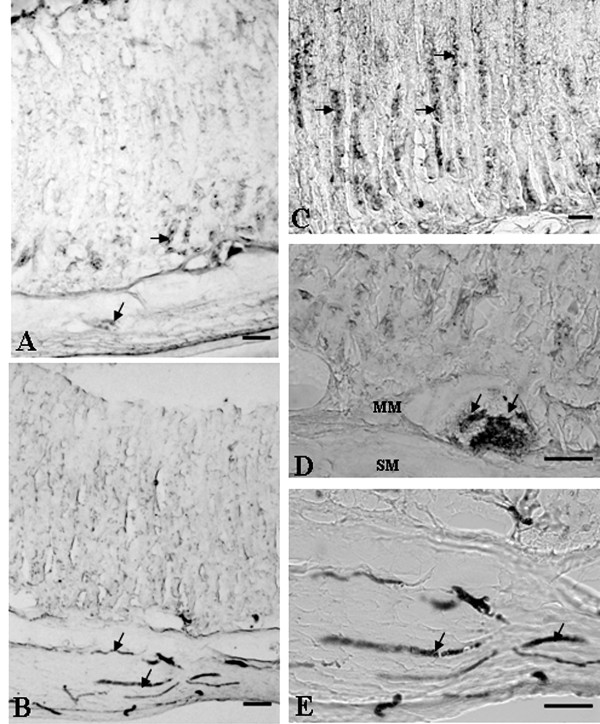
**Immunohistochemical localization of taurine in the rat stomach**. (A) Light micrography of cross-section showing taurine-positive processes in the antrum. (B) Light micrography of a cross-section showing taurine-immunoreactive processes in the body. (C), (D) A higher magnification of the area in (A) showing taurine-positive processes in the antrum. (E) A higher magnification of the area in (B) showing taurine-positive processes in the body. Taurine-immunoreactive cells mostly occurred in glands of the muscle layers. MM, muscularis mucosa; SM, submucosa. Arrowheads indicate taurine-positive processes. Bar = 50 μm.

Numerous myenteric ganglia scattered in the smooth muscle layers of the rat stomach were CSAD positive. CSAD-fibers were to run in muscle layers and in the deep in the muscle layer. CSAD-positive fibers were concentrated in the myenteric plexus and submucosal plexus (Figure 1A and 1D). In the mucosal layers numerous CSAD-immunoreactive cells could easily identified in the deep mucosal layers (Figure 1B and 1C). In addition, numerous taurine-positive myenteric ganglia and fibers distributed all over the muscle layers of the rat stomach (Figure 2). CSAD- and taurine-immunoreactive cells were observed along the length of the mucosal gland (Figure 1C and Figure 2C). No immunoreactive cells were found when non-immune serum was replaced CSAD or taurine antibody.

### Acid Secretion

**Figure 3:**
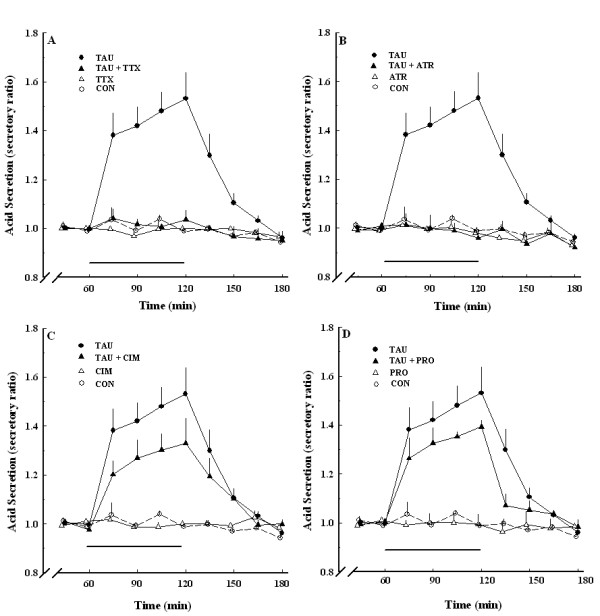
**Taurine-induced acid secretion in the absence and presence of TTX (A), atropine (B), cimetidine (C), and proglumide (D)**. TAU, 10^-6 ^M taurine alone (●, *n *= 6); CON, control (○, *n *= 6); TTX, 3 × 10^-7 ^M TTX alone (Δ, *n *= 6); TAU+TTX, 10^-6 ^M taurine and 3 × 10^-7 ^M TTX (▲, *n *= 6); ATR, 10^-6 ^M atropine alone (Δ, *n *= 6); TAU+ATR, 10^-6 ^M taurine and 10^-6 ^M atropine (▲, *n *= 6); CIM, 10^-6 ^M cimetidine alone (Δ, *n *= 6); TAU+CIM, 10^-6 ^M taurine and 10^-6 ^M cimetidine (▲, *n *= 6); PRO, 3 × 10^-4 ^M proglumide alone (Δ, *n *= 6); TAU+PRO, 10^-6 ^M taurine and 3 × 10^-4 ^M proglumide (▲, *n *= 6). Each point represents the mean ± SEM.

Spontaneous acid secretion reached a steady state after equilibration for 2 h. The average spontaneous acid secretion after equilibration was 1.232 ± 0.067 μmole/15 min, which was taken as the control value. Taurine did not affect the spontaneous acid secretion at 10^-6 ^M (Figure 3). The taurine (10^-6 ^M)-induced acid secretion was completely inhibited by TTX at 3 × 10^-7 ^M and atropine at 10^-6 ^M (Figure 3A and 3B). A histamine H_2_-receptor antagonist, cimetidine, at 10^-6 ^M and an antagonist for the gastrin receptor, proglumide, at 3 × 10^-4 ^M, did not significantly affect taurine at 10^-6 ^M-induced acid secretion (Figure 3C and 3D).

**Figure 4:**
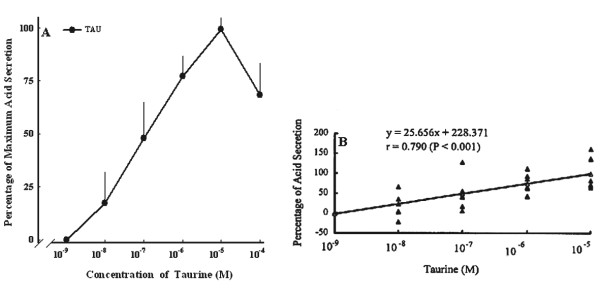
**Effect of various doses of taurine-induced acid secretion in the isolated stomach**. (A) Dose-dependent curve of taurine-induced acid secretion. (B) Correlation between various doses of taurine and acid secretion. Values are the mean ± SEM (*n *= 6).

Taurine at 10^-9^~10^-4 ^M increased the acid secretion in a concentration-dependent fashion, and the ED_50 _value for taurine was 1.2 × 10^-7 ^M. The maximum acid secretion occurred as taurine at 10^-5 ^M with a secretory ratio of 1.6 (*n *= 6) (Figure 4A). Taurine increased acid secretion in the stomach in a dose-dependent manner. Taurine concentration highly correlated (*r *= 0.795, *p *< 0.001) with acid secretion (Figure 4B).

**Figure 5:**
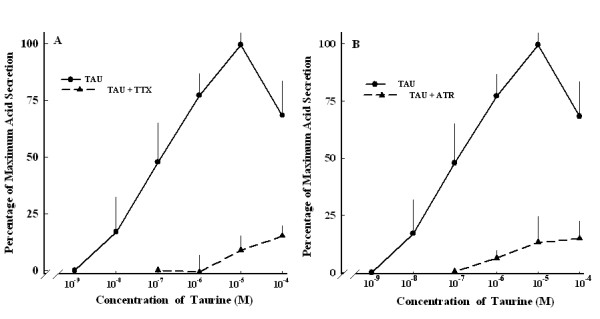
**Dose-dependent curve of taurine-induced acid secretion with and without atropine (A) and TTX (B)**. TAU, taurine alone (●, *n *= 6); TAU+ATR, taurine and 10^-6 ^M atropine; (▲, *n *= 6). TAU+TTX, taurine and 3 × 10^-7 ^M TTX; (▲, *n *= 6). Each point represents the mean ± SEM.

TTX at 3 × 10^-7 ^M abolished the acid secretion induced by taurine at ≤ 10^-6 ^M, but did not completely inhibit induction by taurine at > 10^-6 ^M (Figure 5A). Atropine, a muscarinic receptor antagonist, completely inhibited the acid secretion induced by taurine at ≤ 10^-7 ^M, but only a certain extent of the secretion induced by taurine concentrations > 10^-7 ^M (Figure 5B). The TTX-insensitive component was < 15% of the response obtained by taurine at ≥ 10^-6 ^M and was similar to the atropine-insensitive component.

**Figure 6:**
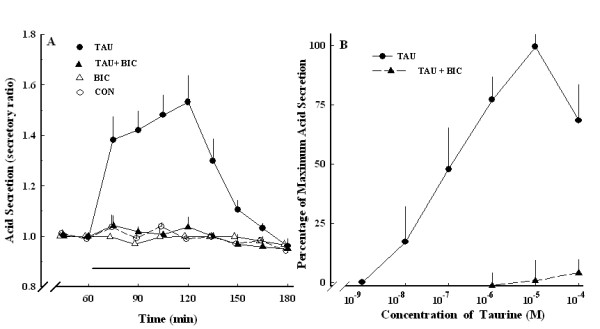
**Effect of taurine-induced acid secretion in the absence and presence of bicuculline**. (A) Acid secretion expressed as the secretory ratio was plotted against the time duration expressed in minutes. (B) Effect of 10^-6 ^M bicuculline on various concentrations of taurine-induced acid secretion. TAU, taurine alone (●, *n *= 6); CON, control (○, *n *= 6); TAU+BIC, taurine and 10^-6 ^M bicuculline (▲, *n *= 6); BIC, 10^-6 ^M bicuculline alone (Δ, *n *= 6). Data are the mean ± SEM.

Bicuculline (10^-6 ^M), an antagonist of the GABA_A _receptor, produced a concentration-dependent decrease in taurine-induced acid secretion at 10^-9^~10^-4 ^M. Bicuculline at 10^-6 ^M abolished the acid secretion induced by taurine at ≤ 10^-6 ^M, but did not completely inhibit induction by taurine at > 10^-6 ^M (Figure 6). Acid secretion was not affected by baclofen, an agonist of the GABA_B _receptor (data not shown).

**Figure 7:**
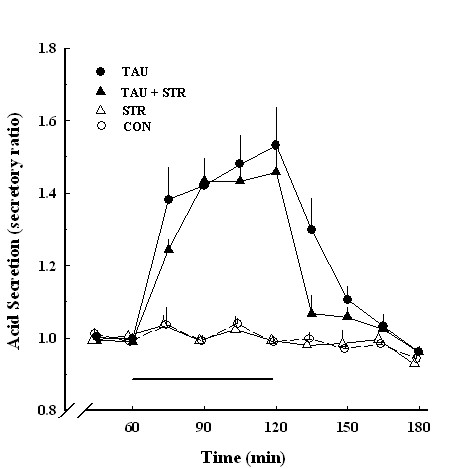
**Effect of taurine-induced acid secretion in the absence and presence of strychnine**. Acid secretion expressed as a secretory ratio was plotted against the time duration expressed in minutes. TAU, 10^-6 ^M taurine (●, *n *= 6); CON, control (○, *n *= 6); STR, 10^-6 ^M strychnine (Δ, *n *= 4); TAU+STR, 10^-6 ^M taurine and 10^-6 ^M strychnine (▲, *n *= 4). Data are the mean ± SEM.

Strychnine, a glycine receptor antagonist, did not significantly affect taurine-stimulated acid secretion at 10 ^-6 ^M (Figure 7).

**Figure 8:**
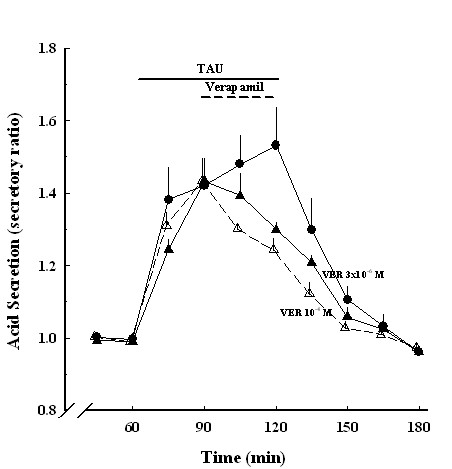
**Effects of taurine-induced acid secretion in the absence and presence of verapamil**. Acid secretion expressed as secretory ratio was plotted against the time duration expressed in minutes. TAU, 10^-6 ^M taurine (●, *n *= 6); TAU+VER, taurine and 10^-4 ^M verapamil (Δ, *n *= 6); TAU+VER, 10^-6 ^M taurine and 3 × 10^-6 ^M verapamil (▲, *n *= 6). Data are the mean ± SEM.

Verapamil, a calcium blocker agent, had no effect on spontaneous acid secretion, but after 3 h, the secretion rate began to decrease (data not shown). Verapamil (3 × 10^-6^~10^-4 ^M) significantly decreased taurine (10^-6 ^M)-induced acid secretion (Figure 8).

### Measurement of the cAMP Concentration

**Figure 9:**
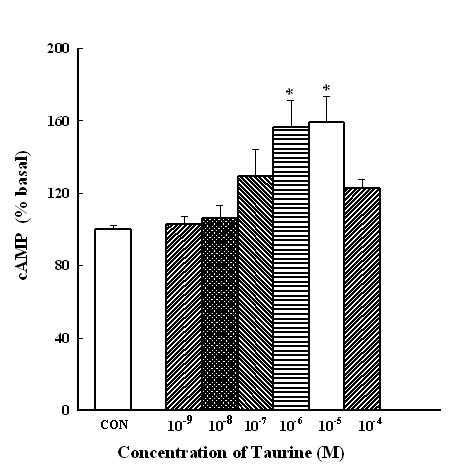
**Effects of various concentrations of taurine on cyclic nucleotide levels in mucosal slices of the rat stomach**. Samples were incubated at 37°C for 30 min before the addition of taurine were treated within 2 min for the production of cAMP. Each column represents the mean ± SEM of the percent basal level. * *p *< 0.05, significantly differs from the control (C) group (*n *= 5).

The gastric mucosa was cut into slices and bubbled in a solution with a mixture of 95% O_2 _and 5% CO_2 _at 37°C in water bath incubation for 2 min. The spontaneous cAMP concentration was 1.673 ± 0.223 pmole/mg protein. Taurine at 10^-9^~10^-4 ^M stimulated increases in the intracellular cAMP concentration in the stomach slice in a dose-dependent manner. Therefore, taurine (10^-6 ^M) markedly increased the cAMP concentration to 100-156% in the stomach slice (Figure 9).

## Discussion

In this communication we further support the notion that taurine may play an important role in the stomach. First, taurine markedly increases gastric acid secretion. Second, taurine stimulates acid secretion that abolished by TTX, atropine, and bicuculline but not by cimetidine, proglumide, or strychnine. Third, taurine potently increases the level of cAMP. Fourth, the presence of taurine-containing cells in the rat stomach is confirmed, as indicated by CSAD- and taurine-positive cells.

We found the presence of taurine-containing cells and taurine-induced acid secretion in the stomach. In the body of the stomach, taurine-immunoreactive cells were observed along the length of the mucosal gland. It had been reported that taurine protects the gastric mucosa from damage caused by monochloramine [33]. Therefore, taurine stored in the mucosal glands may protect cells from self-destruction during oxidation. Taurine-containing cells are present in the myenteric plexus and submucosal plexus of the enteric nervous system in the stomach. Taurinergic neurons in the muscle layer of the gastrointestinal (GI) tract might be involved in motility of the GI tract and the function of endocrine cells as well.

Taurine at 10^-6 ^M markedly stimulated acid secretion in the stomach. Spontaneous acid secretion from the preparation was 1.232 ± 0.067 μmole/15 min, a value similar to the basal acid secretion in vivo [34] and in vitro [22]. In such preparations, taurine induced acid secretion in a concentration-dependent manner. Therefore, taurine acts not only on the CNS [10,35-37] but also on the stomach itself to induce acid secretion.

The parietal cells apparently possesses specific receptors for histamine, gastrin, and acetylcholine (ACh) [38]. We found that cimetidine and proglumide had no significant effect on the taurine-induced acid secretion. This suggests that histamine and gastrin may not participate in these events.

TTX completely inhibited the acid secretion induced by low taurine concentration ≤10^-7 ^M but did not completely inhibit the acid secretion induced by high taurine concentration >10^-7 ^M. It's been long recognized that low TTX concentrations blocks nerve conduction due to inhibition of the Na^+ ^channel [39]. The inhibitory effect can be attributed blocking nerve conduction. Atropine completely inhibited acid secretion induced by low taurine concentrations ≤ 10^-7 ^M. Therefore, the neuronal pathway involved in acid secretion induced by low taurine concentrations may predominantly involve in cholinergic neurons. Both TTX and atropine completely inhibited the acid secretion induced by low concentrations of taurine (10^-7 ^M and under). In contrast, there two components in the acid secretion induced by high concentrations of taurine (10^-6 ^M or above). The component insensitive to atropine was to much the same degree as that insensitive to TTX. Whether taurine at high concentrations induces acid secretion by direct action on parietal cells or by indirect actions on other cells remains to be determined.

Taurine is a general agonist for all types of receptors, e.g., GABA_A _receptors and glycine receptors [10,40]. Taurine has multiple functions in the brain by participating in both modulation and neurotransmission. Taurine-induced acid secretion was inhibited by bicuculline, an antagonist of the GABA_A _receptor. Strychnine (10^-6 ^M), a glycine receptor, did not inhibit taurine-induced acid secretion in the stomach. Recently, pharmacological studies have found that taurine binds to GABA receptors [24-26]. There is compelling evidence that taurine interacts with the GABAergic system via the GABA_A _receptor [24,41-43]. Taurine as also been shown to activate a taurine receptor [44] or through the glycine receptor [45], but the molecular identity of this receptor has not been fully characterized. Yet, studies also indicate that taurine-produced effects can not be simple function. It is interesting that taurine has also been shown can bind to GABA receptors in the rabbit [46] and the mouse brain [47] but not pig brain [44]. Thus, different animal species and studies models may produce different results. In the present investigation, taurine can increase acid secretion via the A type of GABA_A _but not GABA_B _and glycine receptors in the rat stomach.

Gastric acid secretion is not only stimulated via the classical known neuronal and hormonal pathways but also by the Ca^2+^-Sensing Receptor (CaSR) located at the basolateral membrane of the acid-secretory gastric parietal cell. More recent studies have shown that in addition to these well described receptors a CaSR has been identified and is active in acid-secretory parietal cells [48-50]. Previous investigation found that verapamil, an inhibitor of L-type Ca^2+^-channels reduced stimulation suggesting that both the release of intracellular Ca^2+ ^from the ER as well as Ca^2+ ^influx into the cell are involved in CaSR-mediated H^+^/K^+^-ATPase activation [48]. Thus, verapamil to block Ca^2+^-influx from the extracellular space could cause the inhibition of taurine-induced acid secretion.

In addition, taurine effectively increases cAMP concentration in stomach by binding to GABA_A _receptors on cholinergic neurons, resulting in the excitation of cholinergic neurons, followed by the release of ACh. The ACh-binding M_3 _receptors exist on the membranes of parietal cells. Extracellular Ca^2+ ^appears to be an important factor in the control of gastric secretion [51].

## Conclusions

Our results demonstrated for the first time in taurine-induced acid secretion due to increase intracellular calcium may act through the A type of GABA receptors, which are mainly located on cholinergic neurons though cAMP pathway and partially on nonneuronal cells in the stomach. In light of the findings of previous investigations together with our observations of CSAD- and taurine-positive cells in the stomach and taurine released from CSAD- and taurine-containing neurons, which is also consistent with the above hypothesis.

If peripheral taurine is involved in modulating gastric function is on way of investigation.

## Competing interests

The authors declare that they have no competing interests.

## Authors' contributions

This study was designed and supervised by RHL and LHT. Experiments were performed by KHH and CCC. Analysis of the data was performed by KHH, CCC and JDH. LHT drafted the manuscript and all authors read and approved the final version.
